# How the COVID-19 pandemic signaled the demise of Antarctic exceptionalism

**DOI:** 10.1126/sciadv.adk4424

**Published:** 2024-03-01

**Authors:** Daniela Liggett, Bob Frame, Peter Convey, Kevin A. Hughes

**Affiliations:** ^1^ University of Canterbury, Christchurch, New Zealand.; ^2^ British Antarctic Survey, Cambridge, United Kingdom.; ^3^ University of Johannesburg, Johannesburg, South Africa.

## Abstract

This paper explores how the COVID-19 pandemic affected science and tourism activities and their governance in the Antarctic and Southern Ocean. The pandemic reduced the ability of Antarctic Treaty Parties to make decisions on policy issues and placed a considerable burden on researchers. Tourism was effectively suspended during the 2020–2021 Antarctic season and heavily reduced in 2021–2022 but rebounded to record levels in 2022–2023. The pandemic stimulated reflection on practices to facilitate dialog, especially through online events. Opportunities arose to integrate innovations developed during the pandemic more permanently into Antarctic practices, in relation to open science, reducing operational greenhouse gas footprints and barriers of access to Antarctic research and facilitating data sharing. However, as well as the long-term impacts arising directly from the pandemic, an assemblage of major geopolitical drivers are also in play and, combined, these signal a considerable weakening of Antarctic exceptionalism in the early Anthropocene.

## Teaser

The COVID-19 pandemic unsettled Antarctic science and tourism operations and weakened the long-held perceptions of exceptionalism.

## INTRODUCTION

The Coronavirus Disease of 2019 (COVID-19) pandemic was neither unexpected nor unprecedented, although media rhetoric suggested otherwise. COVID-19 can be regarded as one of three major challenges to society globally in the early 21st century, after the 2001 terrorist attacks of “9/11” and the global economic crisis and recession in 2007. The pandemic has been identified as the worst global public-health crisis in a century (*1*), and a growing body of scholarly literature is reporting on its effects on various aspects of complex and dynamic socioecological systems (*2*).

The academic literature already highlights that the effectiveness of responses to the pandemic depended on the tactics used, the level of resourcing made available by governments, and on the compliance of their citizens (*3*, *4*). Multi-institutional and international cooperation has been required to manage the adverse economic, political, sociocultural, public health, and environmental impacts of a pandemic that has affected livelihoods and human activities. The remotest corners of Earth have not escaped the pandemic’s impacts, including the Antarctic, the region of our particular interest. The area south of 60°S latitude, covering 10% of Earth’s surface and devoid of Indigenous human populations, was set aside for peace and science by the 1959 Antarctic Treaty, which entered into force in 1961. The Treaty and its related agreements, collectively known as the Antarctic Treaty System (ATS), regulate international relations with respect to the Antarctic. Parties to the Treaty meet annually at the Antarctic Treaty Consultative Meetings (ATCM) to make governance decisions. The Antarctic’s remoteness and biophysical as well as other practical barriers to access serve to make the region potentially one of the more controllable international spaces regarding the spread of a pandemic.

While isolated, Antarctica is visited by growing numbers of people engaged in science and science-support operations, tourism, and associated undertakings as well as commercial fishing. All visitors to the Antarctic originate from, and travel through, other regions en route to the continent. The Antarctic landmass experienced a first confirmed incursion of the severe acute respiratory syndrome coronavirus 2 (SARS-CoV-2) as early as December 2020 (note that we refer to SARS-CoV-2 when referring to the virus itself and to COVID-19 when emphasizing the phenomenon of infections with SARS-CoV-2 and their impacts), while the virus is almost certain to have already been taken to Antarctic waters via infected passengers or crew on board the tourist vessel M/S *Greg Mortimer* in March 2020, with multiple cases developing on board before any subsequent calling points during its return from Antarctica (*5*).

In this paper, we explore how the COVID-19 pandemic played out in Antarctica and unsettled decision-making processes. In addition to the impact of the pandemic on Antarctica and Antarctic operations (*6*–*11*), the responses to these changes inform how the Antarctic discourse is changing globally. Drawing on international research efforts by the Antarctic community across the biophysical sciences, the social sciences and the humanities under a project supported by the Scientific Committee on Antarctic Research (SCAR), we utilize an extensive body of work to discuss how the pandemic has affected human engagement with the Antarctic. In particular, we examine the implications of the pandemic on the science and tourism sectors within which most human activities in the Antarctic are situated (including logistical support for both) and on their overall governance through the ATS. We also make passing reference to the impact of the pandemic on the fisheries sector.

We examine how the Antarctic’s geographic isolation and its consensus-based international governance regime have affected responses to the COVID-19 pandemic. We assess the role of coordination and collaboration in a science sector with a long history of collaborative endeavor, and the level to which the challenges posed by the pandemic have been responded to, from individual researchers to major institutions. We conclude by discussing the extent to which the pandemic unsettled the dominant discourses in science and, to a lesser extent, tourism, in parallel with a broader assemblage of other geopolitical drivers that have increased tensions in the Antarctic and globally. This required looking at broader geopolitical shifts, especially within the most influential Treaty Parties, and increasing protectionism within the Antarctic spheres of activity. This aligns with growing concern more generally about the condition of the ATS (*12*–*14*). For example, Mancilla and Jabour conclude with a prognosis that, “if not severely ill, the [Antarctic Treaty] system’s chronic ailments—particularly laggardness—must be addressed if it is to respond satisfactorily to rapid social, political, environmental and economic changes on a global scale” (*14*).

In the following sections we, first, provide some contextual background on the Antarctic generally and for this study specifically. Then, we discuss the various impacts and responses to the pandemic before concluding with an exploration of what these developments mean for the Antarctic.

### Context: Antarctic exceptionalism

The Antarctic is one of the world’s most remote regions, surrounded by the cold and inhospitable Southern Ocean. It is the only continent without a permanent human population and is one of four global commons recognized in international law that cannot presently be subjected to undisputed sovereignty by any nation state. There have been two main historical eras of human engagement with the Antarctic: the “Heroic Age” of exploration starting in the late 19th century and the “Scientific Era” starting in the 1950s at the cusp of the late Holocene/early Anthropocene—with the latter referring to the period during which human activity has become the dominant influence on climate and the environment globally. Here, we take the Anthropocene as an informal concept: a means of highlighting proliferating negative human global impacts and fostering interest in Earth systems dynamics across multiple disciplines, not just geology alone (*15*). The global environmental changes observed in the Anthropocene are one of the main areas of research focus in the Antarctic, and so the relationships between the science communities and the Antarctic governing institutions are both unique to the continent and of global importance.

Antarctica is governed under the ATS, an international consensus-based regime established in the midst of Cold War tensions, which devoted the Antarctic continent and the surrounding ocean south of 60°S latitude to peace and scientific cooperation. Over the course of more than half a century, the Antarctic Treaty has grown into a body of agreements that regulate human activities in Antarctica and the Southern Ocean. The latter is the focus of the Convention for the Conservation of Antarctic Marine Living Resources (CAMLR Convention), which was agreed in 1980 and entered into force in 1982 and is one of the subsidiary agreements that sits within the ATS but that effectively operates independently of the ATCM. While following a precautionary ecosystem management approach, it is widely considered in practical terms to be a regional fisheries management organization. Any human activities in the Antarctic (other than fishing) are regulated by other instruments within the ATS, of which the Antarctic Treaty itself and the Protocol on Environmental Protection to the Antarctic Treaty, which was agreed in 1991 and entered into force in 1998, are arguably the most important. Science and scientific collaboration have been recognized as a key raison d’être for human presence in the Antarctic and are considered to represent the currency of Antarctic diplomacy (*16*).

SCAR, one of the affiliated bodies of the International Science Council, plays a substantial role in the international facilitation and coordination of Antarctic research. SCAR has currently 47 member countries, including all the Consultative Parties to the Antarctic Treaty and non-Treaty nations such as Luxembourg, Thailand, Mexico, and Iran. SCAR is entirely independent of the ATS and plays a dual role, whereby its other function is to provide impartial and independent scientific advice to the ATCM, either by request or proactively. The Council of Managers of National Antarctic Programs (COMNAP) represents the national Antarctic programmes of 32 states, and it is those programmes that enable and support Antarctic field research and scientific programmes. The sources of funding for the scientific research vary across nations but do not generally come from the programmes themselves but from national research councils or equivalent bodies. SCAR, COMNAP, and the Commission for the Conservation of Antarctic Marine Living Resources (CCAMLR), which gives effect to the CAMLR Convention’s objectives and principles, are the only organizations with observer status at the now annual ATCMs, during which the Parties discuss and make decisions on matters relating to human activities in the Antarctic and how these are managed. Only Consultative Parties have voting rights on decisions, but both Consultative Parties and Observers can table Working Papers, which are introduced and discussed at the ATCMs. Non-Consultative Parties neither have voting rights nor can they table Working Papers, unless they copropose a Working Paper jointly with a Consultative Party. All participants to an ATCM can submit Information Papers that can, but do not have to, be discussed in the meeting (*17*).

The way the Antarctic is governed and mediated as a cultural space and place (*18*) has notably set it apart from all other regions of the globe, including the other three global commons (the high seas, the atmosphere, and outer space). The Antarctic Treaty Consultative Parties (ATCPs) have repeatedly emphasized Antarctica’s special status and have treated it as an exceptional region (*19*). This concept of Antarctic exceptionalism has not only been applied to Antarctic governance but has also been drawn on by the media to paint a picture of a cold, remote, desolate, and pure place in the far south (*11*). While scholars have argued more recently that environmental challenges, such as the climate crisis, and global political and financial challenges have eroded Antarctic exceptionalism by underscoring the interconnected and interdependent character of all parts of the world, the media in various countries have continued to reinforce Antarctica’s position as an exceptional place, especially after the onset of the COVID-19 pandemic (*11*). This echoes the description in 1911 of Antarctica as “the only almost germ-free continent left” by the explorer Douglas Mawson, which has remained as cultural perception of the region up to and including the pandemic (*20*). Leane *et al.* (*20*) argue that the role of pandemics and viruses has been part of the cultural perceptions of Antarctica over the past century. They propose that the recent media construct of Antarctica during the pandemic needs to be understood against this context (*20*). With this background, we now explore the unsettling impacts of the pandemic on those involved with Antarctic science, tourism operations or governance, their responses to its impacts, and how, alongside other drivers of change, the pandemic is a harbinger of a shift in the overall Antarctic discourse marked by the loss of Antarctic exceptionalism. An erosion of Antarctic exceptionalism in the Anthropocene has already been observed in Antarctic governance (*21*) and broader sociocultural systems (*22*), but the impacts of the COVID-19 pandemic on the Antarctic and Antarctic institutions provide further evidence of declining Antarctic exceptionalism.

### The arrival of SARS-CoV-2 in the Antarctic

For most of the first year of the COVID-19 pandemic, the Antarctic was portrayed predominantly in the light of the absence of SARS-CoV-2 and hailed as a last bastion of pristine, virus-free space (*23*). However, the eventual ingress of COVID-19 was practically inevitable, given continued movement of people through multiple countries to and from the continent. It first occurred at the very end of the 2019–2020 Antarctic summer season (October to April) in what had been a record-breaking year for Antarctic tourism (*24*). The virus was detected aboard the Motor Vessel (M/V) *Greg Mortimer* (*9*, *23*), a tourist vessel which, as a self-contained space, and as seen in multiple other cruise liners around the world at the time, acted as an effective viral incubator. Of those on board the vessel, 59% of its 217 passengers and crew eventually tested positive for SARS-CoV-2, and one crew member died by the time the vessel, which had not been allowed access to Ushuaia in southern Argentina as per its original return itinerary, was eventually permitted to anchor offshore in Uruguay. The ensuing plight of the passengers and crew from the vessel and their arduous and long journeys back to their respective home countries were representative of the stress experienced by cruise tourists and seafarers alike. Many were stranded either at sea or in countries that had permitted them to disembark but without readily available opportunities to cross international borders and return home (*25*). This case also highlights the difficulty of obtaining reliable information from authorities.

In December 2020, the virus was first recorded at an Antarctic station, when maintenance workers at the Chilean Bernardo O’Higgins Research Station (in the northwest Antarctic Peninsula) were confirmed to have been infected (*10*, *11*, *23*), with 58 cases eventually being reported. This marked the first official SARS-CoV-2 infections announced by a national Antarctic programme and represented an important milestone for the Antarctic continental landmass, which had, to that point, been free of the virus. Because of many national Antarctic programmes essentially putting most of their field programmes on hold during the 2020–2021 season and focusing on doing the bare minimum to keep their year-round Antarctic research stations functioning, the continent was spared the initial notable rise of COVID-19 infections and mortality seen elsewhere in the world. COVID-19 reappeared on the Antarctic continent 1 year later, in December 2021, this time at the Belgian research station Princess Elisabeth in remote Dronning Maud Land, where 64% of a group of 25 fully vaccinated researchers tested positive for what was presumed to be the Omicron variant of SARS-CoV-2 7 days into their expedition to the station. Whether other individuals also became infected when they met the researchers as they traveled to the station via South Africa and other Antarctic locations is not known.

SARS-CoV-2 was subsequently recorded at the Argentinean Esperanza Base in the Antarctic Peninsula in January 2022, where nine unvaccinated people of the 24 who had been infected with the virus were evacuated from the station via helicopter. In the 2022–2023 season, the virus arrived on the other side of Antarctica in the Ross Sea region at the U.S. McMurdo Station, which can host up to 1200 personnel, making it the continent’s largest research station. The arrival of the virus resulted in the largest COVID-19 outbreak on the continent to date. By November 2022, approximately 10% of the science-support personnel and researchers at McMurdo had been infected with SARS-CoV-2, effectively causing station life and planned research projects to be put on hold. Additional cases were recorded in November 2022 at the French Dumont d’Urville Station in Adélie Land, where 20 of 21 station staff tested positive, and in January 2023 at New Zealand’s Scott Base, which is located 3 km from McMurdo Station on Ross Island, among other confirmed cases on national operator ships and stations in this period.

No COVID-19–related deaths have been reported to date by national Antarctic programmes, possibly due the initial proactive decisions to adopt an elimination strategy with regard to SARS-CoV-2 and to cut their activities to a minimum in the first year of the pandemic, followed by the subsequent introduction of coordinated mitigation and prevention mechanisms. On the basis of risk assessments, as the global situation evolved and as vaccines became more readily available and immunity or resistance increased, COMNAP moved from an elimination strategy to a mitigation strategy, which involved accepting that SARS-CoV-2 would occur in Antarctica but managing the risks arising from the presence of the virus via required vaccinations and quarantine protocols. This change in management approach was considered carefully, taking into account the particulars of the Antarctic situation (*26*).

When SARS-CoV-2 emerged in Antarctica, it caused substantial disruption to scientific research and Antarctic logistics including station activities. Had these infections occurred while the more virulent earlier variants of SARS-CoV-2 were circulating, the consequences would have probably been more serious. Medical prevetting of station and science personnel traveling to the continent with national Antarctic programmes, and their typically younger and statistically less vulnerable demographic than the general population, may have reduced the likelihood of national Antarctic programmes having to deal with the most serious symptoms of the virus. Nevertheless, symptoms, pathogen transmission and patient management can become further complicated in the Antarctic as:

1) Extreme dryness aggravates viral respiratory infections;

2) the intensely compact nature and communal-living arrangements make effective isolation of anyone infected with SARS-CoV-2 near-impossible (*9*);

3) there are no intensive care facilities in the Antarctic, and limited numbers of qualified medical personnel with severely restricted availability of medical evacuation operations, which are also critically dependent on weather and light conditions and generally not feasible over winter (*9*); and

4) unanswered questions about procedures around engaging support and identifying evacuation routes through multiple operators or countries potentially further limit evacuation capacity and effectiveness (*9*).

In addition to the disruptions that SARS-CoV-2 caused for scientific programmes and station operations, its arrival adversely affected international station inspections and monitoring. A lack of necessary inspections is likely to result in less transparency with regard to operations and may also mean that operational shortcuts or inefficiencies with adverse environmental impacts, or transgressions in environmental management processes, are overlooked. Last, the risk of zoonotic transfer from infected humans or via migratory species to Antarctic wildlife exists, although COMNAP assessed this risk as low. To date, this transfer has only been theorized and has not been shown to have occurred (*6*, *27*).

### Impacts of the COVID-19 pandemic

Human activities in the Antarctic are predominantly science- and tourism-related, although there are also extensive fishing operations in the Southern Ocean. Activities undertaken by governments or government agencies encompass both logistics, such as national Antarctic programme operations, including the maintenance of field stations and other assets (e.g., runways and other transport infrastructure, camps, depots and refuges, as well as aircraft and vessels) and governance activities, such as inspections and oversight activities as required by the Antarctic Treaty, the Protocol on Environmental Protection to the Antarctic Treaty, or the CAMLR Convention. Access is largely channeled through the gateway cities of Ushuaia in Argentina, Punta Arenas in Chile, Cape Town in South Africa, Hobart in Australia (and to a lesser extent Fremantle), and Christchurch/Lyttleton in New Zealand (*7*). We note that Stanley in the Falkland Islands/Islas Malvinas also offers support services and a stopover port for national vessels en route from Europe and the United Kingdom to the Antarctic Peninsula, and for tourist vessels following a standard circuit from southern South America, through the Falkland Islands/Islas Malvinas, to South Georgia, the South Orkney Islands and the Antarctic Peninsula. During the COVID-19 pandemic, government policies in these gateway countries provided substantial barriers to entry to the Antarctic through closing borders and reducing travel across their sovereign territories. Strict border closures were adopted by New Zealand and Australia, thereby reducing access to the East Antarctic and Ross Sea regions. Similar initial restrictions in the South American gateway countries led to some national Antarctic programmes scrambling for solutions to overcome the difficulties they encountered with regard to getting Antarctic personnel back home at the end of the 2019–2020 Antarctic season, resulting in extended delays and convoluted routes to their home countries, in some cases with enforced extended ship transfers from the Antarctic to home ports (e.g., to the United Kingdom, Norway, and Russia/Belarus). At the end of the 2020–2021 season, foresight and planning meant that national Antarctic programmes had been able to charter ships or aircraft to allow their work to continue and ensure the transfer of station personnel, scientists, and equipment to and from the Antarctic, although the circuitous routings and, in some cases, the continued reliance on long-haul ship transport stretched the perseverance and resilience of personnel. This, in addition to Antarctic-specific factors, such as extreme weather and light conditions, tested the various national Antarctic programmes’ abilities to extricate their personnel from Antarctic stations at the ends of both the 2019–2020 and 2020–2021 seasons. Especially in the first year of the pandemic, various countries faced major challenges transporting their people and cargo, both through sudden loss or change in planned support from other operators, and from the initial near-complete closure of international travel options. We note that very different challenges arose for those in situ in the Antarctic during the pandemic who had to deal with additional psychological stressors related to amended operating procedures, including predeparture quarantine (*10*, *28*), concerns about families and dependents in home countries, those whose Antarctic field seasons had been curtailed or canceled altogether (*29*), or those who were affected by the pandemic in other ways such as, e.g., lost earnings from Antarctic tourism operations nearly grinding to a halt in the 2020–2021 season (*23*).

Access to South American gateway ports (Punta Arenas and Ushuaia) is key for around 20 national operators for logistic support to the Antarctic Peninsula region, where approximately 50% of Antarctic stations and human activities are concentrated, and more widely. At the onset of the pandemic, and in the 2021–2022 season, access was notably reduced, and this was compounded by extended quarantine requirements. For example, the British Antarctic Survey (BAS) lost access to the Antarctic via South America for two full Antarctic seasons and was forced to transport all (2020–2021), or a large proportion (2021–2022), of its personnel (2021–2022) to or from the United Kingdom by ship. This added around 3 months to the typical season length of those personnel and generated personal and family-related challenges as well as severely limiting the numbers of personnel whose seasons could be supported. All BAS ship and aircraft operations in 2021–2022 were directed through the Falkland Islands/Islas Malvinas. They also lost the support of the U.K. government’s naval vessel, HMS *Protector*, through a lack of internal consistency between quarantine and operational requirements required of BAS personnel and naval/governmental personnel traveling on the vessel. These instances posed considerable challenges to the effective continuation of long-term Antarctic research projects and notably reduced the capacity for international scientific collaboration and may continue to do so.

At least five national Antarctic programmes (Finland, the Netherlands, Peru, Portugal, and Sweden) paused their activities altogether in the first season following the outbreak of the pandemic (2020–2021), and overall operational support in that season was only about 40% of the originally planned activity.

### Impacts on Antarctic science

Many Antarctic research and field operations have been delayed by at least 1 to 2 years and some considerably longer, with some field projects canceled entirely (*30*). These delays and cancellations have been further exacerbated by funding cuts (*29*), themselves in part a consequence of the notable economic costs of responding to the pandemic, as well as the large station reconstruction programmes now being carried out by several countries. Together, these produce substantial downstream impacts on access to facilities and the ability to carry out Antarctic science and produce the resulting outputs. Furthermore, they will, in all likelihood, affect development of informed Antarctic governance, which relies on robust and up-to-date scientific knowledge and which we examine further in the next section. In addition, longitudinal studies and funded field research projects that have had already 1- to 3-year enforced delays may suffer negative impacts, not only due to potential future decisions on cancellations or further delays to fieldwork and operations but also due to:

1) loss, including early or scheduled retirement, of experienced staff;

2) the inability to give early career researchers the field training and experience vital to underpin careers in polar research; and

3) predictable funding cuts in response to the economic crises resulting from the pandemic.

These three overarching factors exacerbate the already apparent consequences of over a decade of “flat cash” funding regimes (i.e., annual cuts by the rate of inflation) that have been in place for science in some Western countries (e.g., the United Kingdom) since the global economic crisis of 2008 (*31*). For instance, also in the United Kingdom, a resulting sharp cut in the national Official Development Assistance budget has led to the loss of various aspects of the research undertaken under the umbrella of the BAS, among much wider negative impacts within the non-Antarctic U.K. overseas research community.

A recent study highlighted that the pandemic exacerbated existing inequalities about, for instance, access to research funding or field support (*29*). Some established researchers—mainly those without dependants to care for—reported a short-term increase in productivity during COVID-19 lockdowns when they did not have to spend time traveling to and from work, while “working from home” also allowed a greater opportunity for some to focus on writing in the absence of many of the other normal activities and obstructions characteristic of the workplace. For example, there was a notable increase in Antarctic publications during the COVID pandemic lockdown period 2020–2021. From the end of 2020 to the end of 2021, Antarctic paper output increased by 7.7% compared to only 2.8% for papers produced globally. The high level of output in 2021 would most likely be the longtail publication of papers that were being written and submitted to journals in the preceding year. The same pattern was not observed in the global pool, which showed a larger increase in output in 2019, before the lockdown period. Conversely, 2022 Antarctic paper output slumped by 10.8% compared with 2021 paper output, which was substantially greater than the 3.9% drop in global paper output, indicating meaningful impact of the pandemic on Antarctic researchers (*29*).

However, for many others, particularly women and those with child-caring responsibilities, the pandemic has increased stress levels; reduced productivity, security, and support; and resulted in substantial mental-health challenges (*29*). The added pressure arising from the COVID-19 pandemic is likely to have widened the gender and age-gap that already existed in the Antarctic scientific community (*9*, *32*).

The pandemic has proved particularly devastating for Antarctic early-career researchers, 85% of whom reported that their work had been negatively affected (*29*). Aside from immediate negative effects of the pandemic on mental well-being and impaired access to supervision or laboratories, early-career researchers also emphasized that the inability to access their field sites, deliver outputs according to their research or funding milestones, loss of income, and the need to find other employment to sustain their livelihoods put their future careers in academia at risk (*29*). Similar negative career outlooks and challenges resulting from the pandemic have been reported by early-career researchers working in other parts of the world and across a wide range of disciplines (*33*). The challenges resulting from the pandemic and its aftermath may also have catalyzed early retirement decisions in the cohort of senior and experienced researchers approaching the later stages of their careers, although evidence supporting this is currently limited (*31*).

Technology has facilitated surrogate social connection during the pandemic, and while Antarctic researchers expressed their support of and appreciation for the availability of online conferences and workshops (*29*), from a mental, social, and societal perspective online interactions are not as healthy or as efficient or effective as meeting face-to-face (*34*), although we note that, for certain people, in-person gatherings can be equally ineffective, awkward, or challenging. Moreover, international online conferences require global participants to work across multiple time zones. However, it is also worth considering that, aside from reducing carbon emissions at a time of climate crisis, virtual events have the benefit of bringing many participants to the table who may not have been able to afford participating in an in-person conference, thereby making some contribution toward leveling the scientific playing field (*34*).

### Impacts on Antarctic governance

Before the pandemic, Antarctic governance and decision-making was already facing major challenges. Externally, there were pressures from outside the ATS, notably climate change and the increasing growth of tourism. Internally, there were pressures resulting from longer-term growth in ATS membership, with recent Parties introducing a broader range of interests and motivations, the limitations of a consensus-based system and the lingering issue of unresolved sovereignty questions (*35*). Assessments of how the ATS is addressing these diverse challenges have not been positive, and the governance regime has been criticized for, inter alia:

1) chronic slowness to respond and a lack of urgency with regard to how existing regulations are implemented (*14*);

2) deficiencies in protecting biodiversity or ecosystems in both the terrestrial and marine realms (*36*); and

3) being slow to address and implement a coordinated response to global environmental change in an Antarctic context (*21*, *37*).

These criticisms raise questions around shortcomings of the regime with respect to its integrity, accountability, moral acceptability, benefit sharing, and, ultimately, its legitimacy (*12*, *38*, *39*). The pandemic exacerbated these challenges, especially regarding the slowness to respond to issues such as climate change (*8*).

The 2020 ATCM, which had been scheduled to take place 2 to 3 months after the start of the pandemic, was canceled due to the practicalities of organization at that very uncertain time. Concerns were raised that the cancellation of the 2020 ATCM may have reduced the international accountability of the regime (*40*) and may also have long-term consequences (*7*, *8*).

Hosted by France, the 2021 ATCM was held entirely online and was shorter than normal, focusing only on urgent issues to catch up on the cancellation of the 2020 ATCM. In 2022, an in-person meeting in Berlin, Germany, resumed, but a hybrid option was offered to accommodate representatives unable to attend due to specific travel restrictions, including the then ongoing lockdown in China and the illegal Russian invasion of Ukraine (*16*). The latter represents an enormous challenge to the ATS and its consensus-based decision-making processes. Both Ukraine and Russia have voting rights in the ATS as Consultative Parties and are members of CCAMLR. Before 2022, no Consultative Party had ever invaded the sovereign home territory of another Consultative Party. The resulting stress and conflict were palpable at the 2022 ATCM and unsurprisingly caused frictions that extended beyond these two Parties during the meeting, which are likely to persist (*41*). At the 2022 ATCM, and specifically covering issues related to the pandemic, COMNAP stated that “national Antarctic programmes working together to respond to the COVID-19 challenge might have been the greatest example of international collaboration in relation to Antarctic activities that it had witnessed in recent times” (*42*).

Despite the challenges faced by the Parties at these aforementioned meetings and the implications they have for Antarctic governance, it is also appropriate to note that the 2023 ATCM took the unusual step of setting aside an entire day for a joint session with CEP, and included SCAR and COMNAP, to consider the implementation of the recommendations in SCAR’s Antarctic Climate Change and the Environment report (*43*). The Meeting encouraged Parties and Observers to bring experts, who are not normally involved in ATCM processes, to the meeting to support this work (*44*). A small number of scientific presentations set out the current realities and future threats of climate change in the Antarctic, but much of the day was spent preparing the “Helsinki Declaration on Climate Change and the Antarctic” (*45*), and it remains unclear what longer-term benefit will result. In contrast, CCAMLR held a workshop on climate change in September 2023, which included contributions from many Parties and resulted in a workshop report with 25 recommendations that were subsequently endorsed by the CCAMLR Scientific Committee in October 2023.

The 2020 CCAMLR meeting went ahead as a shortened four-hour virtual meeting (*46*), resulting in far fewer topics being tabled than normal, with discussions curtailed, which has been, more generally, linked to a potential loss of transparency and equity in decision-making (*46*). A further downside of the virtual meeting was that critical decisions were postponed to 2021 (*46*). On the flipside, shifting to a virtual space resulted in cost savings for CCAMLR (*47*) and allowed less well-off national delegations and observers to increase their participation. In international context, moving the CCAMLR meeting online was not an exception but a practical necessity which had then increasingly become the rule (*48*, *49*) and may have been inevitable at the time. The 2022 and 2023 CCAMLR meetings were held in person in Hobart, Australia. However, returning to in-person meetings does not seem to have re-invigorated the Commission, which, at its most recent meeting, failed to move forward proposals for further environmental protection in the form of three additional Marine Protected Areas that had been first received in 2022 (*50*).

Overall, a reduced focus on Antarctic governance during the pandemic had major implications for decision-making and regulatory oversight of the Antarctic continent and in the Southern Ocean. Virtual meetings required emergent ways of working, which may result in a permanent change in the way CCAMLR, the ATCM, and/or the Committee for Environmental Protection, which is a key advisory body to the ATCM, undertake elements of their business. Traditional in-person gatherings provide the opportunity to address controversial issues in the margins of the meeting through hallway discussions and unofficial interactions. Virtual meetings do not facilitate these informal discussions, which probably inhibited progress during the virtual ATCM and CCAMLR meetings. Some ATCM and CCAMLR meeting participants commented on how problematic certain aspects of the online meetings were and how they had become less effective. However, even in prepandemic times, meetings of, e.g., CCAMLR, had become increasingly both politicized and factionalized and simply blaming that the meeting format for a lack of effectiveness may not be entirely appropriate (*51*). Potentially, this politicization could lead to a wider erosion of the effectiveness of the ATS and add to questions about its role as the region’s governing body.

### Impacts on Antarctic tourism

For tourism, the extremely negative global consequences of lockdowns, travel restrictions, infection rates, and illness during the COVID-19 pandemic are well documented (*52*) and also include consequential impacts on the citizen science and science and logistic support offered by the tourism industry. Antarctic tourism was no exception, with operations essentially ceasing in the 2020–2021 season (*10*, *23*), with only 15 visitors on two yachts (one of those a scientific expedition) venturing into Antarctic waters. In the 2021–2022 season, Antarctic tourism numbers increased to 22,979 passengers on 235 voyages (*53*, *54*), which was slightly over half of the number of tourists visiting the Antarctic in the 2019–2020 season when 54,485 passengers on 367 voyages were recorded (*24*). This temporary pandemic-enforced reduction in tourism to the Antarctic that adversely affected the sector not only as an immediate consequence but also in the longer term has led to a loss of guides and experienced staff. Tourism activities resumed with visitor numbers well above prepandemic levels in the 2022–2023 austral summer (*54*), with a total number of 104,076 tourists reported to have visited the Antarctic (*55*).

We also note that, globally, fisheries were affected by the pandemic. While some studies report a positive effect of lockdowns and movement restrictions on fish stocks and the regeneration of some sensitive aquatic ecosystems (*56*), reduced oversight and policing has also given rise to increased illegal fishing activities within and outside marine protected areas (*57*) including the Southern Ocean. In the CCAMLR area, the catch of krill makes up approximately 98% of the biomass of fish caught with almost all caught in CAMLR Convention Area 48. While krill catches have increased steadily in recent years, they are still some way off the 620,000–metric ton total limit for Area 48. Looking at catch levels between 2019 and 2022, the highest catch was during the austral winter of 2020, when the COVID pandemic was at its height and the number of vessels (*5*) was at its highest during 2020, followed by 2022 and 2021 (*58*). While there is some variability in annual krill catch, the data suggest that the COVID pandemic had neither an impact on the upward trend of krill harvesting in the Southern Ocean nor was there an obvious effect on fishing effort by fishing nations (notably Norway with its technologically advanced vessels).

Collective consideration of these impacts of the pandemic points to an unsettling of the science and tourism sectors but not in fisheries. We now seek to investigate the possible implications of this and the benefits that might arise before looking more broadly at what this might mean for the role of science in society globally.

## DISCUSSION

The disruptions observed are now reviewed considering their impacts, both positive and negative, and in the contemporary, swiftly changing and unstable, global geopolitical context. This not only highlights how the pandemic exacerbated existing long-term challenges and introduced emerging ones to the Antarctic system but also points to a considerable weakening of Antarctic exceptionalism.

### Learning from the pandemic

Polar science (both Arctic and Antarctic) experienced a sharp temporary decline as an immediate result of the pandemic (*59*). While research activity levels are now recovering, it is already stated that knock-on delays will continue to affect multiple national operators and the international research community for several more years at least. Furthermore, these already-admitted delays will also be compounded by the increasingly complex synergies with other global financial and geopolitical drivers.

While the Antarctic science community has established mechanisms and discrete national organizations to facilitate rapid and effective coordination, the preparedness of national Antarctic programmes and the approaches taken to preventing or managing SARS-CoV-2 infections in the Antarctic were far from uniform. The range of styles or types of response of different governments to COVID-19 domestically generally framed how national Antarctic programmes dealt with the pandemic in an Antarctic context. In various countries, this national response also depended on domestic political factors, irrespective of resources (e.g., in Brazil, Russia, United Kingdom, United States, etc.), and the effectiveness of any response was driven by how long it had taken for governments to respond in the first place and whether remedial actions were necessary to reverse prior unsound decisions (*60*). In the Arctic, there was no coherent pan-Arctic response, and different parts of the Arctic region fared differently during the pandemic as it was poorly equipped for mass medical emergencies (*59*). Despite the low population density across the Arctic, the impact of COVID-19 there was on a par with the rest of the world (*61*), although cases were not evenly distributed, with the Russian North disproportionately negatively affected as compared to other Arctic regions.

The Antarctic science and tourism sectors were confronted with a situation for which preparations were few or nonexistent and that stimulated action in terms of reviewing institutional processes for the purposes of information sharing and decision-making. This sense of unsettling has been observed consistently throughout the current project’s research. However, it has also provided a rehearsal for subsequent pandemics and, hopefully, important lessons have been gained for effective future handling of these emergencies. In turn, a detailed cost-benefit analysis in relation to how the COVID-19 pandemic has been handled in the Antarctic context would inform more robust responses to future pandemics.

### Responding to the pandemic globally and in Antarctica

Science and tourism in Antarctica operate within global systems, which were also disrupted by the pandemic, and these transnational implications provide a wider context for Antarctic operations.

For researchers, the impact globally (*62*) and for field researchers (*63*) is well documented and similar to that experienced by those involved in the Antarctic. Existing inequities and gender disparities in publications widened during the pandemic (*32*, *64*), with long-term negative implications for women in academia (*65*). A study of 1100 participants in U.S. higher education showed that more than half (55%) considered changing careers or retiring early because of a work landscape that had notably changed because of COVID-19 (*66*). Compared to 2019, anxiety and stress doubled, with females disproportionately affected. COVID-19 “pushed many faculty members to the verge of burnout” (*66*), threatening a higher education landscape that should be diverse and experienced (*66*).

The pandemic had, arguably, some positive impacts especially in health and medical research and globally resulted in more collaboration (*4*), speedier and more open publication processes (*67*, *68*), and more flexibility in terms of quickly adjusting research foci. Interdisciplinarity experienced a boost when, for example, anthropology and social sciences were included in a United Kingdom Research and Innovation emergency response call. Following the pandemic, certain aspects of science in some countries are in higher demand from both policymakers and journalists (*69*).

In terms of climate change and environmental impacts (*70*), the pandemic demonstrated that internationalism, cooperation, and knowledge sharing does work in counteracting global threats. There were, albeit fleetingly, some positive environmental consequences with a temporary decline in carbon emissions and air pollution following cuts in local transport and international travel (*71*, *72*), during which there were instances of temporary environmental restoration (*73*), although we lack solid evidence for this in the Antarctic. An isolated observation in the Antarctic suggests that the reduced number of people visiting or residing in the Antarctic during the pandemic might have had a positive effect on at least some species of penguin. Flynn *et al.* (*74*) noticed a notable increase in gentoo penguin nesting sites around Port Lockroy in the Antarctic Peninsula between 2018 and 2021, which caused them to hypothesize that ship and vessel traffic might cause more disturbance for these species than previously thought. Similar observations on the effects of the anthropause on species or ecosystems have been made elsewhere (*75*) but are neither generalizable nor yield convincing evidence that any positive effects will last or, in fact, that they are systemic.

Transformative technologies and changes in organizational practices helped to overcome physical distancing and facilitate both operational and policy exchanges, which are likely to remain both globally and in terms of Antarctic processes. We “live in a never-ending transformation process, in which how we live, relate, and speak with others has been modified for all time” (*76*). The pandemic encouraged scientific communities “to step outside of our comfort zone and embrace changes that are, in some cases, long overdue” (*63*) and encourage more efficient and effective use of technologies to facilitate research collaborations (*63*), data sharing, and remote observations (*77*). Solutions adopted during the pandemic, such as online meetings replacing in-person conferences, are only short-term fixes in terms of the climate crisis.

### Potential responses to challenges

Lessons for societies globally and the Antarctic science and tourism sectors emerge from the literature on how the COVID-19 pandemic affected humankind and how states, communities, organizations, and individuals responded. Scerri *et al.* (*63*) have summarized these as key areas for urgent innovation that coincide with our earlier work (*29*). These areas for innovation are opportunities that have already shaped some responses to challenges that existed before the pandemic or since its inception can be thematically categorized:

1) science-policy interactions to be privileged through more open science with identifiable pathways and incentives for societal benefits,

2) fieldwork and operations to be decarbonised through accountable and auditable processes that link to national emissions targets,

3) research processes to be improved with more efficient data sharing and collaboration,

4) research partnerships to be ethical, equitable, and established with direct involvement by parties from the Global South, and

5) communities of practice to reduce barriers to entry to Antarctic science and scientific conferences, especially for Global South participants and states with developing Antarctic programmes.

Acknowledging that all these areas for improvement relate to, and build on, the broader consideration of the importance of just transitions (*78*) to a more sustainable future, a critical question remains about the extent to which these might be viable in the near term and whether they could be tackled by Antarctic communities on their own or in partnership with other Treaty institutions. In particular, the pandemic prompted the Antarctic science community to review their research processes and partnerships and to ensure more efficient sharing of data across institutional and national boundaries, with the goal of, e.g., enabling early-career researchers whose field season was unexpectedly canceled to complete their doctoral or postdoctoral research projects (*29*). Similarly, innovations occurred in the realm of utilizing remote sensing technologies to a greater extent for Antarctic data collection, and SCAR has been prompted to set in motion a review of its carbon footprint, which is work that is presently underway. There are, of course, countering influences, and we now provide a brief indication of the main difficulties.

During the pandemic, while there was little impact on the fisheries sector, the Antarctic science and tourism sectors were confronted by complexities that unsettled their operations, with their special status distinct from elsewhere in the world, including other global commons. The pandemic disrupted these communities, accustomed as they were to operate with relatively secure long timelines and a comparative sense of entitlement to operate in a relatively unconstrained, albeit modest, manner. The pandemic highlighted increasing limitations to Antarctic exceptionalism.

The pandemic was but one driver affecting the Antarctic and human activities in the region, and it cannot be easily isolated from the changing global context. It is beyond the scope of this article to explore the assemblage of global drivers in detail. However, there are two specific drivers that have manifested themselves concurrently with the pandemic, namely, broader geopolitical shifts, and increasing protectionism. Combined, these form an insidious, largely invisible counter to the role of science as envisaged under the ATS. They highlight that, while Antarctica’s role in the global system continues to be, at least in part, unique, the region is simultaneously wholly absorbed by and dependent on the interactions with, and disruptions of, global politics, science, and human engagement within the global commons. Together, they threaten the idea that Antarctic science is divorced from global affairs and largely dissolve the notion of exceptionalism.

### Other drivers of change: The influence of geopolitical shifts on Antarctic operations

Broad geopolitical factors relating to Antarctica, which, while already of concern (*12*, *14*), have accelerated during and since the pandemic. With Russia and Ukraine, both ATCPs, the invasion of Ukraine has affected activities in the Antarctic. Russia, after the collapse of the Soviet Union, took over the ownership and management of all 10 Antarctic stations previously operated by the Union of Soviet Socialist Republics. Ukraine runs the Akademik Vernadsky Station, which was fully staffed from the 2021–2022 through to the 2022–2023 season and is serviced by Ukraine’s icebreaker Research Vessel (R/V) *Noosfera* (previously the United Kingdom’s Royal Research Ship (RRS) *James Clark Ross*), which, after encountering and escaping Russian shelling in the Black Sea, spent much of her time out of Russia’s militaristic reach in the Southern Hemisphere and continues to conduct limited research (*79*). At the 44th ATCM in Berlin, Germany (2022), many Parties criticized the military operations by Russia and the support provided by Belarus (a non-Consultative Party) while emphasizing the peaceful philosophy of the Treaty and the desire that, post-pandemic, the “ATCM’s work for peace, research, and environmental protection should not be compromised because of the aggression of one Party against another” (*80*).

China’s aspirations as a polar superpower (*81*–*83*) include specific aspects of its role in the conservation of marine living resources and governance (*35*). China currently operates five stations and, since the pandemic, has restarted, in early 2023, their construction of a station on Inexpressible Island in the Ross Sea region. In contrast, the U.S. Department of Defense has signaled this expansion of China’s presence in the Antarctic, in combination with China’s increased activity in the Arctic, to be of concern (*84*). They note that China’s increased presence in the Antarctic is “likely intended to strengthen its position for future claims to natural resources and maritime access” (*84*). While this may be a contested issue, this rhetoric in a report to the U.S. Congress underlines China’s increasing influence in the Antarctic. Furthermore, the report highlights that China’s dual-use technologies, facilities, and scientific research are likely intended, at least in part, to improve their military capabilities (*84*). Furthermore, the developing relationship between China and Russia has implications for Antarctica with, for example, their combined reluctance to support proposals for marine protected areas around Antarctica (*85*, *86*). It can also be seen in China’s statements that the ATCM should restrict itself to Antarctic issues and not become involved in the Russia-Ukraine conflict (*80*).

### Other drivers of change: Increased national protectionism in Antarctic governance

Closely associated with geopolitical factors are issues relating to national protectionism. Now, this is most clearly seen by the complexity of the 70 permanent research stations operated by 29 different countries scattered across Antarctica. Globally, surveillance and nationalist isolation, rather than solidarity, were problematic aspects of national responses to the pandemic (*87*). The various approaches taken by different states to local or regional lockdowns and border closures effectively resulted in a geopolitical and geographical “rebordering” of the world. Potentially, this might lead to a rise in protectionist attitudes that may well reduce levels of cooperation, as indicated by some national positions taken at the 2022 ATCM in Berlin (*80*). The utilization of (i) polar research results for strategic (geo)political and even militaristic purposes, and (ii) scientific presence in furtherance of national interests and national identity has received increasing scholarly attention in an Arctic context (*88*). In the Antarctic, science has played a crucial role in facilitating collaboration, diplomacy, and evidence-based decision-making (*16*, *89*). However, science has equally been utilized as an instrument that allows countries to maintain presence in the region and has been considered a stepping stone toward gaining decision-making status in the ATS (*90*). While scientific collaboration is a core value upheld by the Antarctic Treaty with national Antarctic programmes coordinating activities, exchanging information and guidance on best practice, and facilitating scientific and logistical cooperation, flaws in the process are emerging. The global increase in protectionism, acts of rebordering, and geopolitical tensions between some Antarctic Treaty Parties contribute to value chasms opening within the ATS, with the potential to substantially affect the role, impact, and organization of future Antarctic science.

### Final thoughts

Antarctica’s research communities were established as part of the continent devoted to peace and science and renowned for its purity (*20*), but they are, increasingly, subject to the same complex assemblage of drivers of change as the rest of global society. The pandemic serves as a distinct example of this that unsettled Antarctic scientific activities, tourism operations, and policy making, in ways that will take time to fully evolve and understand. While specific innovations and interventions by SCAR, COMNAP, and the wider Antarctic science and tourism sectors innovations have softened some of the impacts of these changes, no simple solution for a swift and improved way of working exists. The fact that SARS-CoV-2 traveled to the Antarctic continent despite its geographical isolation and substantial barriers of access to the region—both managerial and technological—is evidence of an undeniable global connectedness and the intricate entanglement of global environmental and geopolitical system. Antarctica, which has been designated by the Protocol on Environmental Protection to the Antarctic Treaty as a “natural reserve devoted to peace and science” and Antarctic communities have not been spared by the pandemic despite the best efforts by Antarctic decision-makers and operators to keep Antarctica free of the virus. This highlights that the Antarctic cannot be considered as separate from the rest of the world any longer. We conclude that the Antarctic exceptionalism, which blossomed during the scientific era of the late Holocene, is drawing to a close and that the early Anthropocene will see a much more complex interplay with broader global drivers including, but possibly not restricted to, geopolitics and protectionism. Progressing from Antarctic exceptionalism toward more integrated operational and governance processes would appear to be a critical step in accommodating the changes inherent to the early Anthropocene. The pandemic provides evidence that this is achievable, although not necessarily easy.

## MATERIALS AND METHODS

This article is the result of a collaborative content analysis and synthesis of research results emerging from SCAR’s Standing Committee on the Humanities and Social Sciences’ project “The impact of COVID-19 on Antarctica” (*91*). This project addressed two key questions (*91*): (i) What were the impacts of COVID-19 on Antarctic research and researchers? (ii) What are the long-term implications of COVID-19 for Antarctic operations and governance?

This present study primarily builds on quantitative and qualitative data published in over a dozen scholarly articles and one report to the SCAR Executive Committee that resulted from the project’s five work packages that focused on implications and impacts of the COVID-19 pandemic on (i) Antarctic futures, (ii) research and decision-making, (iii) tourism, (iv) public perceptions of Antarctica, and (v) Antarctic wildlife. Furthermore, the present study also draws extensively on a suite of policy documents and formal reports of the ATCM, COMNAP, and CCAMLR, a range of strategic governmental and nongovernmental reports, news articles, and the rapidly growing body of scholarly literature on the pandemic and Antarctic science and tourism.

This article, which syntheses and contextualizes work done under the work packages, which drew on methods used in humanities and social sciences as well as the biological, medical, and veterinary sciences, seeks to “consolidate the results obtained from all other working packages and aims at developing a suite of recommended actions to be taken to mitigate adverse impacts of COVID-19 on Antarctic communities of practice” (*91*). Consequently, this article aligns with interdisciplinary approaches to research which are highly qualitative in nature.

The bibliographic dataset to assess Antarctic-related publication outputs derived from all Antarctic papers from 2013 to 2022 was created from searches in web of Science using the following topic search string, which was taken from Gray and Hughes (*92*): TS = {[antarc* NOT (candida OR “except antarctica” OR “not antarctica” OR “other than Antarctica”)] OR “transantarctic” OR “ross sea” OR “amundsen sea” OR “weddell sea” OR “southern ocean”} AND PY = (2013–2022). The results were benchmarked using InCites to create a custom dataset of Antarctic papers for benchmarking analysis. The InCites dataset used was updated on 27 October 2023 and including web of Science content indexed through to 30 September 2023.
